# A lightweight and secure two factor anonymous authentication protocol for Global Mobility Networks

**DOI:** 10.1371/journal.pone.0196061

**Published:** 2018-04-27

**Authors:** Ahmed Fraz Baig, Khwaja Mansoor ul Hassan, Anwar Ghani, Shehzad Ashraf Chaudhry, Imran Khan, Muhammad Usman Ashraf

**Affiliations:** 1 Department of Computer Science & Software Engineering, International Islamic University, Islamabad, Pakistan; 2 IBMS, Agriculture University Faisalabad, Pakistan; King Saud University, SAUDI ARABIA

## Abstract

Global Mobility Networks(GLOMONETs) in wireless communication permits the global roaming services that enable a user to leverage the mobile services in any foreign country. Technological growth in wireless communication is also accompanied by new security threats and challenges. A threat-proof authentication protocol in wireless communication may overcome the security flaws by allowing only legitimate users to access a particular service. Recently, Lee et al. found Mun et al. scheme vulnerable to different attacks and proposed an advanced secure scheme to overcome the security flaws. However, this article points out that Lee et al. scheme lacks user anonymity, inefficient user authentication, vulnerable to replay and DoS attacks and Lack of local password verification. Furthermore, this article presents a more robust anonymous authentication scheme to handle the threats and challenges found in Lee et al.’s protocol. The proposed protocol is formally verified with an automated tool(ProVerif). The proposed protocol has superior efficiency in comparison to the existing protocols.

## 1 Introduction

**Fig 1:**
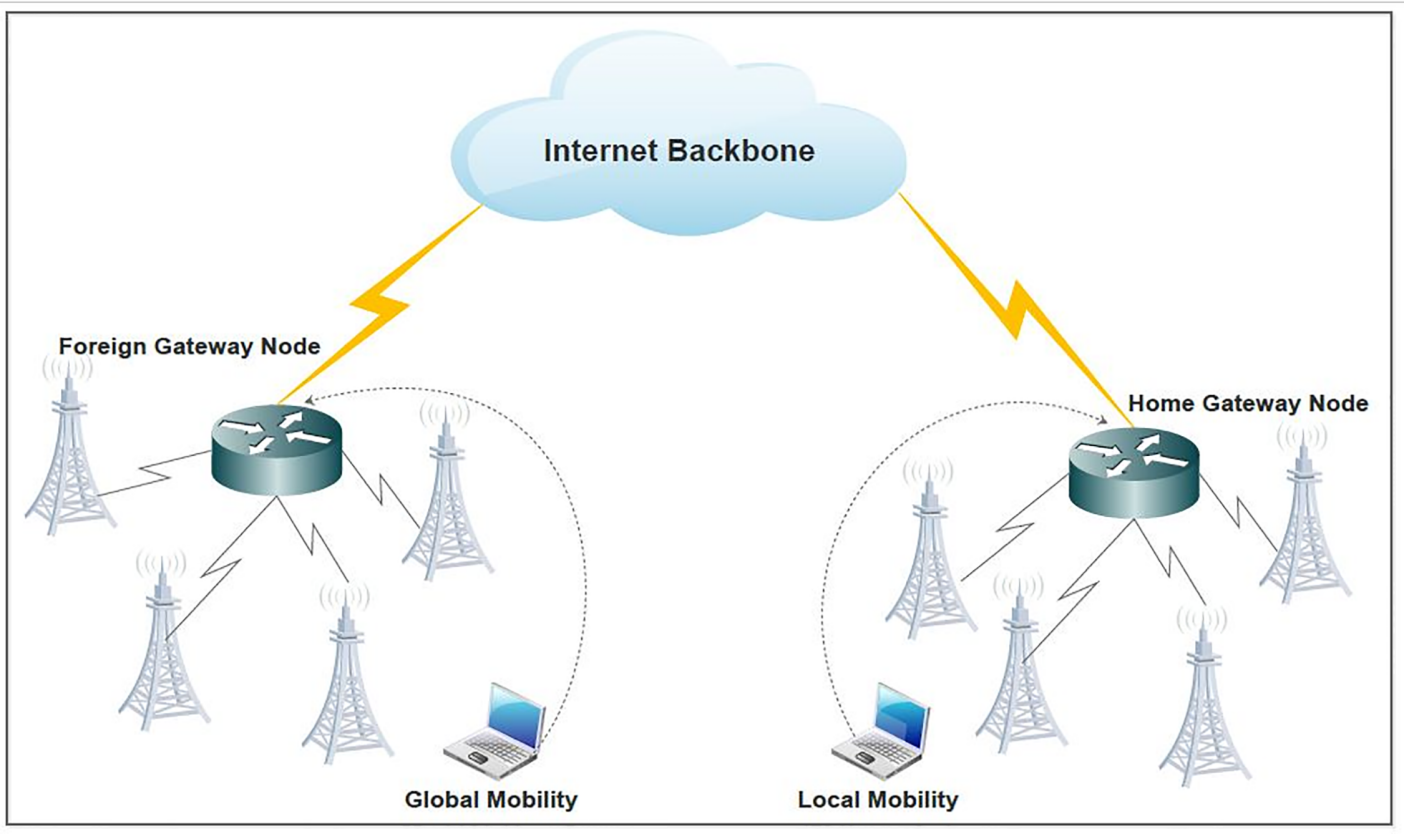
Global mobility networks authentication.

The wireless communications are extensively used in current decade, the internet based applications are accessed by mobile networks at anytime and from anywhere. Nowadays, roaming in mobile communication become extremely famous. Due to the technological improvements many security issues have been raised up because anyone can intercept the communication anytime. While traveling, the mobility services assure that wireless devices are connected with a network without any breakage of connection. When a person visits some other country he/she has to use the mobile services. Global Mobility Networks(GLOMONETs) facilitates a roaming user to leverage their home mobile services in a foreign country [1]. A roaming Mobile Node(MN) uses the mobile services at foreign country with the help of their home country network. Mobile Node(MN) connects to a foreign network in foreign country and Foreign Node(FN) verifies the legitimacy of the Mobile Node(MN) through his/her home network by Home Node(HN)as shown in Fig 1.

Authentication in wireless environment essential and decisive task. Authentication is the only source that ensures the Mobile Node(MN) is a legitimate node [2]. A valid and threat-proof authentication is required for prevention of illegal usage. numerous symmetric, asymmetric and lightweight hash, XOR based authentication schemes are proposed to to provide mutual authentication, node anonymity and to handle different security flaws in GLOMONETs [3–15]. A threat-proof authentication fulfills following requirements: Node anonymity(R1); Node Traceability(R2); Man-in-Middle attack(R3); Backward/Forward secrecy(R4); Replay and Dos attacks(R5); Known-key attacks(R6); Friendliness(R7); Local node and Password verification(R8); Insider attacks(R9); Mutual authentication(R10); Impersonation attacks(R11).

Suzuki et al. [16] in 1997 presented a distributed security based authentication scheme to enable a user to access mobile services in foreign country. Zhu et al. [17] in 2004 presented an authentication protocol that facilitates the features of mutual authentication and implicit mutual secret-key management. Lee et at. [18] disclosed that Zhu et al. [17] scheme is incapable to attain the feature of mutual authentication, moreover, scheme does not resist backward secrecy and impersonation attacks. Lee et al. [18] presented an enhanced authentication protocol to efficiently resolve the imperfections of scheme [17]. Later, the Wei et al. [19]also notified that Zhu et al. [17] scheme inefficient to achieve the user anonymity and also discloses secret information. To overcome these issues the Wei et al. [19] presented a more enhanced protocol that provides secure features like user anonymity and mutual authentication. Wu et al. [20] also found Lee et al. [18] protocol does not achieve the backward secrecy, user anonymity and vulnerable to off-line key guessing attacks. Thus, Wu et al. [20] proposed an efficient protocol that provides resistance of aforementioned attacks. He et al. [21] notified that Wu et al. [20] protocol unable to achieve user anonymity and also vulnerable to replay and forgery attacks. Therefore, He et al. [21] presented a lightweight authentication scheme with the features of strong resistance of stolen verification attacks. Li et al. [22] pointed out He et al. [21] protocol unable to provide the features of user anonymity and also provides unfair key-exchange system. Li et al. [22] presented a protocol that provides the feature of user anonymity and fair key-agreement system. Li et al. [23] pointed out Li et al’s [22] protocol is inefficient due to extra computational cost. Das. [24] also pointed out Li et al. [22] protocol cannot withstand the replay attacks. Yoon et al. [25] presented a new lightweight authentication protocol to handle the loopholes of different protocol with the features of mutual authentication, user friendliness, User anonymity. Niu et al. [26] pointed out the Yoon et al. [25] protocol and proved that protocol does not provide user anonymity and also has an insecure key management system. Therefore, Niu. [26] presented a novel based authentication protocol that provides the feature of user anonymity. Jiang et al. [27] also pointed out that He et al. protocol [21] does not provide strong of two-factor authentication furthermore, the protocol is vulnerable to insider attack, replay attack and failure of user friendliness. The present protocol of Jiang et al. [27] improves the privacy and authentication. Wen et al. [28] proved Jiang et al. [27] protocol does not resist the replay attack and password based verification-attack. Wen et al. [28] presented new protocol that does not enable the users to share the secret-key. Mun et al. [29] presented a new hash and concatenation operation based lightweight scheme. Lee et al. [30] found Mun et al. [29] scheme cannot withstand man-in-the middle attack, masquerade attack and perfect forward secrecy They proposed a more efficient protocol for GLOMNET.

This article notifies that the Lee et al. [30] scheme lacks unfair user registration, inefficient user authentication, unable to provide local user/password verification and vulnerable to replay and DoS attacks.

## 2 Contributions

In this article a detailed analysis of Lee et al. protocol has been presented to check its strengths against various attacks. As a result the following improvements are contributed:

1. Various security weaknesses of the Lee et al. protocol have been identified and elaborated in this paper.
2. A new and lightweight protocol has been proposed in this article which resists different possible attacks and provides the requirement of user friendliness.
3. The proposed protocol has been formally verified using an automated tool “ProVerif” to ensure its security strength.
4. Finally, the proposed protocol has been analyzed for computation and communication efficiency showing better performance than its counterpart protocols.

## 3 Brief review of Lee et al. scheme

Lee et al. presented a lightweight authentication using simple hash operation, XOR and concatenation operations. This section presents precise review of four phases of Lee et al. scheme [30] in following sequence: registration phase, AESK phase, the session key update phase, and the password alter phase. The notation guide is given in Table 1.

**Table 1:** Notations guide.

Notation	Description	Notation	Description
*MN*	Mobile Node	*HN*	Home Node
*FN*	Foreign Node	*T*_*A*_	Timestamps of entity A
*FH*_*k*_	Pre-shared key between *FN* and *HN*	*SK*	Session key
*h*(.)	one way hash function	⊕	The XOR operation
Δ*TS*	Expected time interval for transmission delay	‖	The concatenation operation

### 3.1 Registration phase

The registration phase of Lee et al. scheme is between Mobile Node(MN) and Home Node(HN). The Mobile Node(MN) and Home Node(HN) perform the registration in following steps:

**Step 1:** The Mobile Node(*MN*) chooses {password *PW*_*MN*_, nonce s} and computes EID = *h*(*ID*_*MN*_ ⊕ *PW*_*MN*_) ⊕ *s*. Afterward *MN* forwards a message *M* = {*EID*} to the Home Node(*HN*) over a secure channel.
**Step 2:** The Home Node(*HN*) obtains the message M calculates *S* = *h*(*EID*‖*h*(*SK*_*HN*_)) and sends S to *MN*
**Step 3:** Upon receiving S the *MN* computes *SPW* = *S* ⊕ *h*(*PW*_*MN*_). Finally the *MN* stores *SPW* and s in smartcard(SC).

### 3.2 Authentication and establishment of session-key(AESK Phase)

AESK phase of Lee et al. [30] is performed in following steps:

**Step 1:** *MN* → *FN*: *M*_1_ = {*EID*′, *V*_*MN*_, *Q*_*MN*_, *N*_*MN*_}
  The Mobile Node(*MN*) calculates *EID*′ = *h*(*ID*_*MN*_ ⊕ *PW*_*MN*_) ⊕ *s* and *S*′ = *h*(*EID*‖*h*(*SK*_*HN*_)). MU chooses two nonce *s*_*new*_, *N*_*MN*_. Afterward MN calculates following values $EID_{new}=h\left(ID_{MN}⊕PW_{MN}^{\prime }\right)⊕s_{new}$, *V*_*MN*_ = *EID*_*new*_ ⊕ *h*(*S*′‖*N*_*MN*_) and *Q*_*MN*_ = *h*(*EID*_*new*_‖*S*′‖*N*_*MN*_). Ultimately, a login request message *M*_1_ = {*EID*′, *V*_*MN*_, *Q*_*MN*_, *N*_*MN*_} is forwarded to Foreign Node(*FN*).
**Step 2:** *FN* → *HN*: *M*_2_ = {*EID*′, *V*_*MN*_, *Q*_*FN*_, *N*_*MN*_, *V*_*FN*_, *ID*_*FN*_}
  After receiving the message *M*_1_ Foreign Node *FN* generates a nonce *N*_*FN*_ and calculates *Q*_*FN*_ = *h*(*Q*_*MN*_‖*N*_*FN*_‖*SK*_*FN*_), *V*_*FN*_ = *N*_*FN*_ ⊕ *h*(*SK*_*FN*_). The Foreign Node *FN* sends the message *M*_2_ = {*EID*′, *V*_*MN*_, *Q*_*FN*_, *N*_*MN*_, *V*_*FN*_, *ID*_*FN*_} to Home Node(*HN*)
**Step 3:** *HN* → *FN*: *M*_3_ = {*V*_*HN*_}
  Upon receiving the message *M*_2_ the *HN* computes *S*′ = *h*(*EID*′‖*h*(*SK*_*HN*_)) and afterward computes $EID_{new}^{\prime }=V_{MN}⊕h\left(S^{\prime }∥N_{MN}\right)$ and retrieves $EID_{new}^{\prime }$, after that HN computes *SK*_*FN*_ = *h*(*ID*_*FN*_ ⊕ *SK*_*HN*_), *N*_*F*_ = *V*_*F*_ ⊕ *h*(*SK*_*HN*_). Afterward HN verifies $Q_{FN}\overset{?}{=}h(h(EID_{new}^{\prime }∥S^{\prime }∥N_{MN})∥N_{FN}∥SK_{FN})$ for authentication of Mobile Node(MN) and Foreign Node(FN). Furthermore Home Node(HN) computes *S*_*new*_ = *h*(*EID*_*New*_‖*h*(*SK*_*HN*_), *V*_*HN*_ = (*EID*_*new*_‖*S*‖*S*_*new*_) ⊕ *h*(*SK*_*FN*_‖*N*_*FN*_) and forwards *M*_3_ to FN.
**Step 4:** *FN* → *MN*: *M*_4_ = {*V*_*FN*2_, *Q*_*FN*2_, *N*_*FN*2_}
  Upon receiving *M*_3_, FN derives (*EID*_*new*_‖*S*‖*S*_*new*_) and verifies $Q_{MN}\overset{?}{=}h(EID_{new}∥S^{\prime }∥N_{MN})$ if the verification holds then Foreign Node(FN) authenticates the Mobile Node(MN) and Home Node(HN). Afterward FN generates a nonce and computes *V*_*FN*2_ = *S*_*new*_ ⊕ *h*(*S*‖*N*_*FN*2_), *Q*_*FN*2_ = *h*(*EID*‖*S*_*new*_‖*N*_*FN*2_) and transmits *M*_4_ to Mobile Node(MN).
**Step 5:** Upon receiving the *M*_4_ the Mobile Node(MN) calculates *S*_*new*_ and checks $Q_{FN2}\overset{?}{=}h(EID||S_{new}∥N_{FN2})$ to authenticate the Foreign Node(FN). Afterward the FN updates *SPW*_*new*_ = *S*_*new*_ ⊕ *h*(*PW*_*MN*_) for further use. For a session communication Mobile Node(MN) computes *K*_*FM*_ = *h*(*N*_*MN*_‖*N*_*FN*2_‖*S*), *Q*_*MF*_ = *h*(*N*_*MN*_‖*S*‖*N*_*FN*2_‖*S*_*new*_) and sends *Q*_*MF*_ to Foreign Node(FN) for reconfirmation.
**Step 5:** FN verifies $Q_{MF}\overset{?}{=}h(N_{MN}∥S∥N_{FN2}∥S_{new})$ and computes *K*_*FM*_ = *h*(*N*_*MN*_‖*N*_*FN*2_‖*S*) for the communication of current session.

### 3.3 Session-Key update phase

**Step 1:** The Mobile Node(MN) selects a nonce $N_{MN}^{\prime }$ and calculates *U*_*MN*_ = *N*_*MN*_ ⊕ *h*(*S*‖*N*_*MN*_‖*N*_*FN*2_), $Q_{MN}^{\prime }=h\left(N_{MN}^{\prime }⊕S\right)$ and transmits $Q_{MN}^{\prime },U_{MN}$ to FN
**Step 2:** The Foreign Node(FN) computes $N_{MN}^{\prime }=U_{MN}⊕h(S∥N_{MN}∥N_{FN2})$ and checks $Q_{MN}^{\prime }$. Afterward, FN selects a nonce and calculates $U_{FN}=N_{FN}^{\prime }⊕h(S∥N_{FN2}∥N_{MN}^{\prime })$, $Q_{FN}^{\prime }h\left(N_{FN}⊕S\right)$. Afterward, FN transmits *U*_*FN*_ and $Q_{FN}^{\prime }$ to MN.
**Step 3:** Mobile Node(MN) receives message and calculates $N_{FN}^{\prime }=U_{FN}⊕h(S∥N_{FN2}∥N_{MN})$. Afterward, MN update $K_{FM}^{\prime }=h(N_{MN}^{\prime }∥N_{FN}^{\prime }∥S)$, $Q_{MF}^{\prime }=h\left(N_{MN}^{\prime }⊕N_{FN}⊕S\right)$ and transmits $Q_{MF}^{\prime }$ to FN
**Step 3:** Foreign Node(MN) verifies $Q_{MF}^{\prime }\overset{?}{=}h\left(N_{MN}^{\prime }⊕N_{FN}⊕S\right)$ and updates $K_{FM}^{\prime }=h(N_{MN}^{\prime }∥N_{FN}^{\prime }∥S)$ and completes update phase.

### 3.4 Password alter phase

**Step 1:** Lee et al. scheme enables a Mobile Node(MN) to update his/her password. When a Mobile Node(*MN*) desires to update the password, the Mobile Node(MN) has to login with *ID*_*MN*_ and password *PW*_*MN*_.
**Step 2:** MN uses new password and calculates *EID*_*new*_ = *h*(*ID*_*MN*_) ⊕ *PW*_*New*_) ⊕ *S*_*New*_. Furthermore, for authentication and establishment phase *SPW*_*New*_ is computed and *S*_*New*_ is encrypted with old password *PW*_*MN*_, *SPW*_*New*_ = *S*_*New*_ ⊕ *PW*_*MN*_ and for this phase the new password *PW*_*New*_ is used to encrypt *S*_*New*_, *SPW*_*New*_ = *S*_*New*_ ⊕ *PW*_*New*_. At the end password is altered successfully.

## 4 Security weaknesses of Lee et al. scheme

This section demonstrates the security weakness of Lee et al. scheme [30]. The Lee et al. scheme suffers unfair user registration, inefficient user authentication, vulnerable to replay and DoS attacks furthermore, the Lee et al. scheme does not provide local user and old password verification. The detailed discussion is given in following subsections:

### 4.1 Unfair user registration and inefficient user authentication

The Lee et al. Scheme suffers with a serious flaw in registration phase. The Mobile Node(MN) computes *EID* = *h*(*ID*_*MN*_ ⊕ *PW*_*MN*_) ⊕ *s* and sends *EID* to Home Node(HN) for registration in step1. Whereas, the Mobile Node(MN) takes one way hash(OWH) of *ID*_*MN*_ and password *PW*_*MN*_. When the Home Node(HN) receives registration request message *EID*, the HN would not be able to extract the identity *ID*_*MN*_ form *EID* because there is no such mechanism of de-hashing. Hence, the Home Node(HN) would be unable to recognize user at the registration time and the registration request would be rejected.

In AESK phase of Lee et al. Scheme the Home Node(HN) receives login request through Foreign Node(FN) sent by Mobile Node(MN). The identity of *MN* is saved in *EID*′. To authenticate the Mobile Node(HN) the Home Node(HN) searches for Identity of Mobile Node(MN) in database. Hence, the *ID*_*MN*_ does not exist in Home Node(HN) database and Home Node(HN) cannot recognize the user has sent the login request as a result the Home Node(HN) will reject the authentication request.

### 4.2 Replay and DoS attacks

In Lee et al. Scheme an adversary A will intercept the channel and will obtain login-request message *M*_1_ = {*EID*′, *V*_*MN*_, *Q*_*MN*_, *N*_*MN*_}. As no timestamp or sequence number is associated with login message *M*_1_ the Adv A can replay *M*_1_ in login phase latter on. Likewise the adversary A will perform the replay attacks in step2 with *M*_2_ = {*EID*′, *V*_*MN*_, *Q*_*FN*_, *N*_*MN*_, *V*_*FN*_, *ID*_*FN*_}, step3 with *M*_3_ = *V*_*HN*_ and in step4 with *M*_4_ = {*V*_*FN*2_, *Q*_*FN*2_, *N*_*FN*2_} of authentication phase because any no timestamps or sequence numbers are used with any message. Although, the adversary A is unable to compute the session key but adversary A will send too many login requests intentionally to overwhelm the MN, FN and HN. Simultaneous repetition of replay attacks in large numbers can exhaust the communication and computation cost and also leads to Denial of service(DoS)attacks that may cause the prevention of access the resource to legal user.

### 4.3 Lack of local user and password verification

Lee et al. scheme does not verify old password in phase 5 password alter phase. Any malicious user with a stolen Smartcard(SC) can submit request to change the password. Although the malicious user would not be succeed in this process but He/she can send multiple requests which also lead to DoS as discussed previously. Furthermore, suppose in login phase a Mobile Node(MN) unintentionally, inputs *ID*_*MN*_ and old *PW*_*MN*_. Before transmitting the login request to Home Node(HN) the scheme does not verify the identity ID or password PW are correct or incorrect in login phase. Even if the user enters old password *PW*_*MN*_ for login, the authentication steps(1-4)can still be executed with old ID/PW. Although, at step 4 the Home Node(HN) would reject authentication but this process takes unnecessary computation and communication overhead. Hence, the smartcard(SC) cannot verify the the identity and password of Mobile Node(MN) at login phase which proves inefficiencies in Lee et al. scheme.

## 5 Proposed scheme

Proposed scheme includes of following phases: registration phase, login and authentication phase and password change phase. The detailed description of these phases is as following:

### 5.1 Registration phase

The registration phase of proposed scheme is between Mobile Node(MN) and Home Node(HN). In registration phase the Mobile Node(*MN*) freely chooses an Identity *ID*_*MN*_, password *PW*_*MN*_ and a random number $r\in Z_{n}^{*}$(natural number). Afterward the *MN* computes *U* = *h*(*PW*_*MN*_‖*r*) and transmits a registration request message to *HN* *M* = {*ID*_*MN*_, *U*} on secure channel.

When the Home Node(*HN*) receives the registration request message he/she selects a random number $m\in Z_{n}^{*}$ and computes the following:

$$\begin{array}{ccc} B & = & U⊕h(ID_{MN}∥m) \end{array}$$ (1)

$$\begin{array}{ccc} N_{MN} & = & h\left(U∥R_{T}\right)⊕ID_{MN} \end{array}$$ (2)

Where *R*_*T*_ is the registration time, after that the Home Node(*HN*) stores {*B*, *N*_*MN*_, *m*, *h*(.)} in SC and afterward the smart card(SC) is issued to *MN* through a reliable network channel.

The Mobile Node *MN* regenerates *r* and stores it in smartcard(SC). Now {*B*, *U*, *r*, *h*(.)} are stored in SC database.

### 5.2 Login and authentication phase

**Fig 2:**
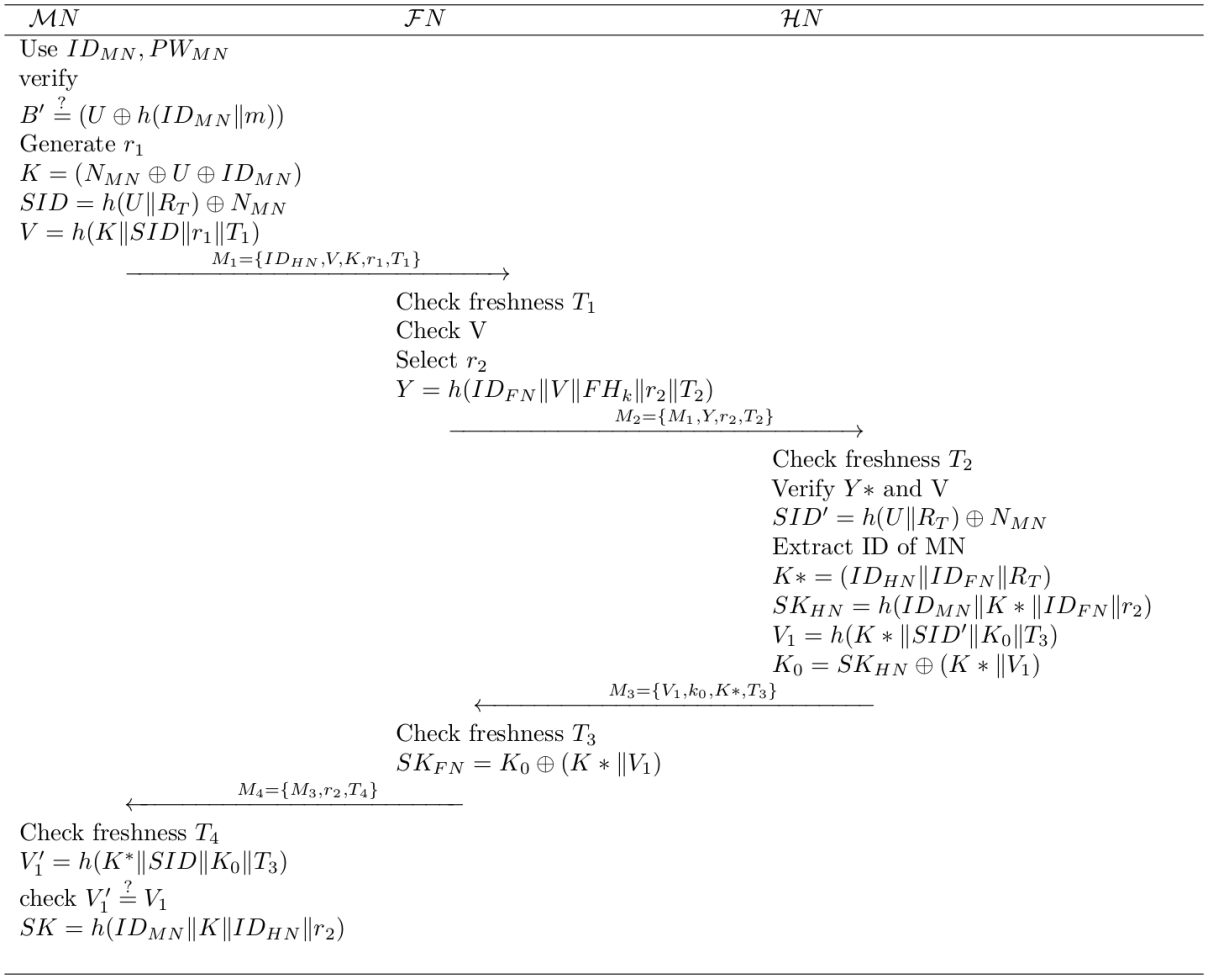
Login and mutual authentication phase of proposed scheme.

For the authentication phases we presume, the Mobile Node(*MN*) is in foreign country under the administration of foreign network. The Mobile Node(MN) intends to use the mobile services in foreign area. To avail the mobile services in foreign region the Mobile Node(*MN*) has to login with Identity *ID*_*MN*_, password *PW*_*MN*_ and afterward for the security and legitimacy he/she will authenticate himself/herself with the help of their Foreign Node(*FN*) and Home Node(*HN*) in a proper manner as shown in Fig 2. After the successful authentication Mobile Node(*MN*) will use the services with collaboration hosted country’s network.

**Step 1:** *MN* → *FN*: *M*_1_ = {*ID*_*HN*_, *K*, *V*, *r*_1_, *T*_1_}
  In first step the user *MN* puts his/her smart card(SC) into the machine and uses his/her identity *ID*_*MN*_ and password *PW*_*MN*_ for login, on login request the machine calculates *B*′ = *U* ⊕ *h*(*ID*_*MN*_‖*m*) that was saved at the registration phase and afterward *MN* compares whether $B^{\prime }\overset{?}{=}B$ if no then session is terminated and login request is rejected. If both B’ and B are same then the legality holds. The smartcard(SC) chooses random number *r*_1_ and calculates the following:
  $$\begin{array}{c} K=(N_{MN}⊕U⊕ID_{MN}) \end{array}$$ (3)
  $$\begin{array}{c} SID=h\left(U∥R_{T}\right)⊕N_{MN} \end{array}$$ (4)
  $$\begin{array}{c} V=h(K∥SID∥r_{1}∥T_{1}) \end{array}$$ (5)
  Where, *T*_1_ is timestamp of Mobile Node(MN). Ultimately, *MN* sends login request message *M*_1_ to Foreign Node(*FN*) over a public channel.
**Step 2:** *FN* → *HN*: *M*_2_ = {*M*_1_, *Y*, *r*_2_, *T*_2_}
  After receiving the message *M*1 Foreign Node(FN) checks the freshness of *T*_1_ if the comparison fails, *FN* does not accept the login request. Afterward Foreign Node(FN) generate a nonce *r*_2_, and calculates the following equations:
  $$\begin{array}{c} Y=h(ID_{FN}∥V∥FH_{k}∥r_{2}∥T_{2}) \end{array}$$ (6)
  Where, *FH*_*k*_ is a pre-shared key between FN and HN. Afterward Foreign Node(*FN*) transmits the *M*_2_ to Home Node(*HN*).
**Step 3:** *HN* → *FN*: *M*_3_ = {*V*_1_, *k*_0_, K*, *T*_3_}.
  When *HN* obtains *M*_2_, the Home Node *HN* confirms the freshness of timestamp *T*_2_ and afterward, verifies both values $V^{\prime }\overset{?}{=}V$ and $Y*\overset{?}{=}Y$ if comparison do not match, the Home Node(HN) rejects *M*_2_ and terminates the session. Afterward Home Node(HN) generates a nonce *r*_3_ and compute following values:
  $$\begin{array}{c} SID^{\prime }=h\left(U∥R_{T}\right)⊕N_{MN} \end{array}$$ (7)
  SID′=h(U∥RT)⊕h(U∥RT)⊕IDMNSID′=IDMNK*=h(IDHN∥IDFN∥RT) (8)
  $$\begin{array}{c} SK_{HN}=h(\left(ID_{MN}\right)∥K*∥ID_{FN}∥r_{3}) \end{array}$$ (9)
  $$\begin{array}{c} V_{1}=h(K*∥SID^{\prime }∥K_{0}∥T_{3}) \end{array}$$ (10)
  $$\begin{array}{c} K_{0}=SK_{HN}⊕\left(K*∥V_{1}\right) \end{array}$$ (11)
  When the Home Node verifies all step then *M*_3_ = {*V*_1_, *k*_0_, K*, *T*_3_} is sent to Foreign Node(*FN*).
**Step 4:** *FN* → *MN*: *M*_4_ = {*M*_3_, *r*_2_, *T*_4_}
  When *FN* obtains message *M*_3_, he/she confirms the freshness *T*_3_ if freshness fails the *FN* rejects the message, otherwise the Foreign Node(*FN*) computes the following equations:
  SK=(K0⊕(K*∥SID∥V′)=SK⊕(K*∥SID∥V′)⊕(K*∥SID∥V′)=SK (12)
  After that for further processing the message *M*_4_ = {*M*_3_, *r*_2_, *T*_4_} is transmitted to Mobile Node(*MN*).
  Upon receiving the message *M*_4_ The Mobile Node *MN* confirms the freshness of *T*_3_ if timestamp is fresh then checks $V_{1}^{{}^{\prime }}\overset{?}{=}h(K^{*}∥SID^{\prime }∥T_{3})$ if the resultant values do not match, then the Mobile Node(MN) terminates the session. Otherwise authentication procedure is completed by Foreign Node(*FN*) and Home Node(*HN*). Afterward, for further communication the Mobile Node(*MN*) computes the session key as following in equation:
  $$\begin{array}{c} SK=h(ID_{MN}∥K∥ID_{HN}∥r_{2}) \end{array}$$ (13)

### 5.3 Password change phase

The password change phase makes the scheme user friendly and enhances the security of the proposed scheme. Our proposed scheme allows the user to update or change their password. Whenever the Mobile Node(*MN*) requests to change the password he/she has to perform the following steps:

**Step 1:** Proposed scheme allows the user to Alter or update the password. When a user with a smartcard(SC) wants to change the password. The user has to login with his/her identity $ID_{MN}^{\prime }$ and enters the password $PW_{MN}^{\prime }$ and performs following steps:
**Step 2:** On the request the smartcard(SC) executes and verifies the following steps:
  $$\begin{array}{c} U^{*}=h\left(PW_{MN}^{\prime }∥r^{\prime }\right) \end{array}$$ (14)
  After the calculation of U* smart card checks whether $U*\overset{?}{=}U$. If the values of U* and *U* are not same then SC reject the request otherwise, it requests the Mobile Node(MN) to choose another new password *PW*_*new*_.
**Step 3:** The Smartcard(SC) calculates the following equations:
  $$\begin{array}{c} B*=(U*⊕h\left(PW_{new}∥m\right)) \end{array}$$ (15)
  N*_MN_ = *h*(*ID*_*MN*_‖*ID*_*HN*_‖*R*_*T*_ ⊕ U*) where, {*B*, *U*, *N*_*MN*_} are replaced with {B*, U*, N*_MN_} and smartcard(SC) carries {B*, U*, *r*′, *h*(.)}.

## Security analysis

This section shows the formal and informal security analysis of proposed scheme. We have analyzed formal verification of proposed scheme with automated tool ProVerif and informally analyzed the scheme against different attacks.

### 5.4 Security analysis with ProVerif

ProVerif [31] may be defined as an automated reasoning software tool or verifier, which verifies cryptographic protocols. The ProVerif handles different cryptographic primitives like: Encryption/decryption, MAC, signatures, hash, Symmetric and asymmetric key cryptography and many others [33]. The formal verification of proposed protocol is tested with this tool, the detailed description of code and results are given below.

**Fig 3:**
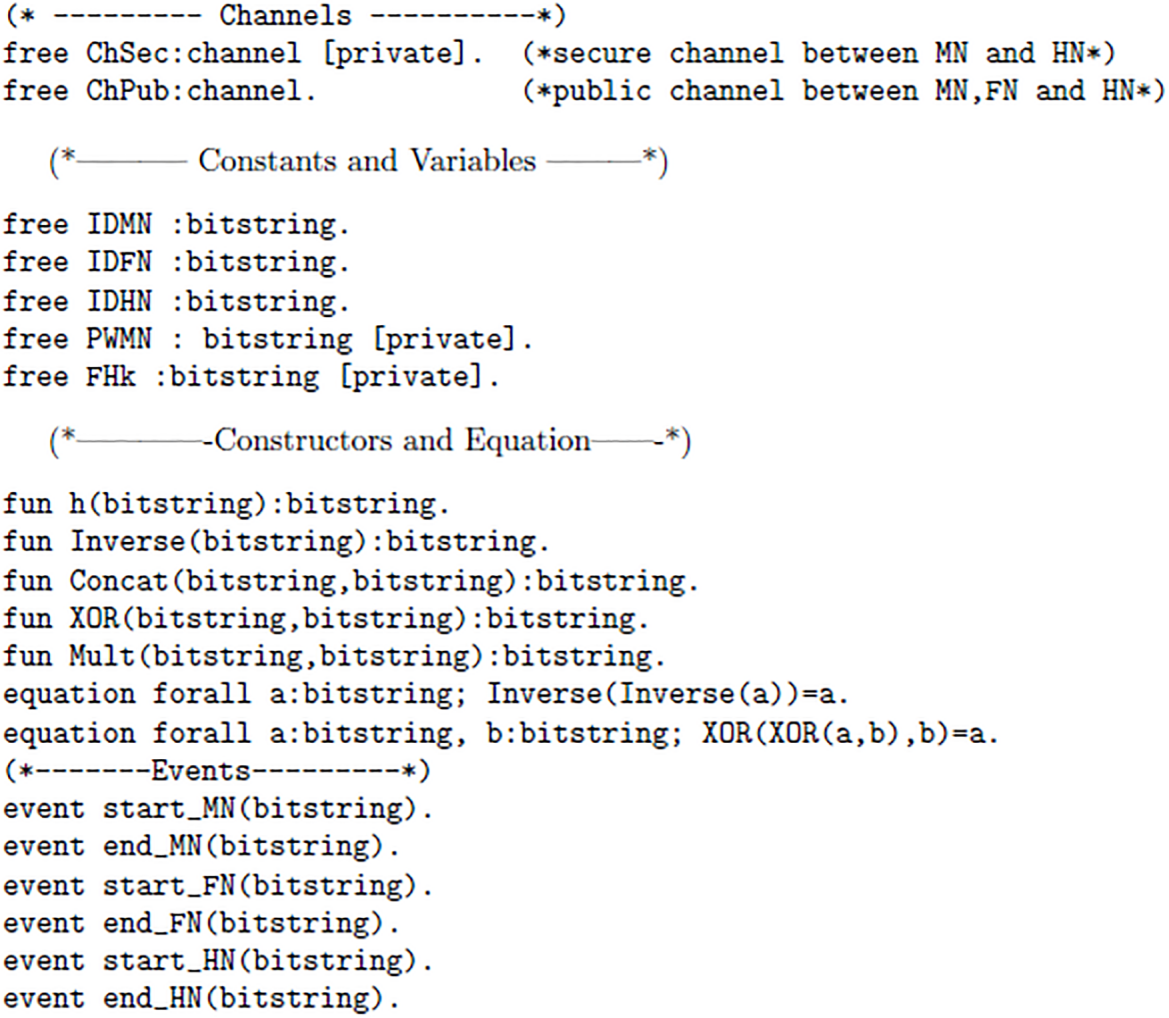
1(a).

**Fig 4:**
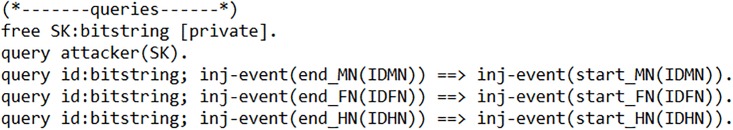
1(b).

**Fig 5:**
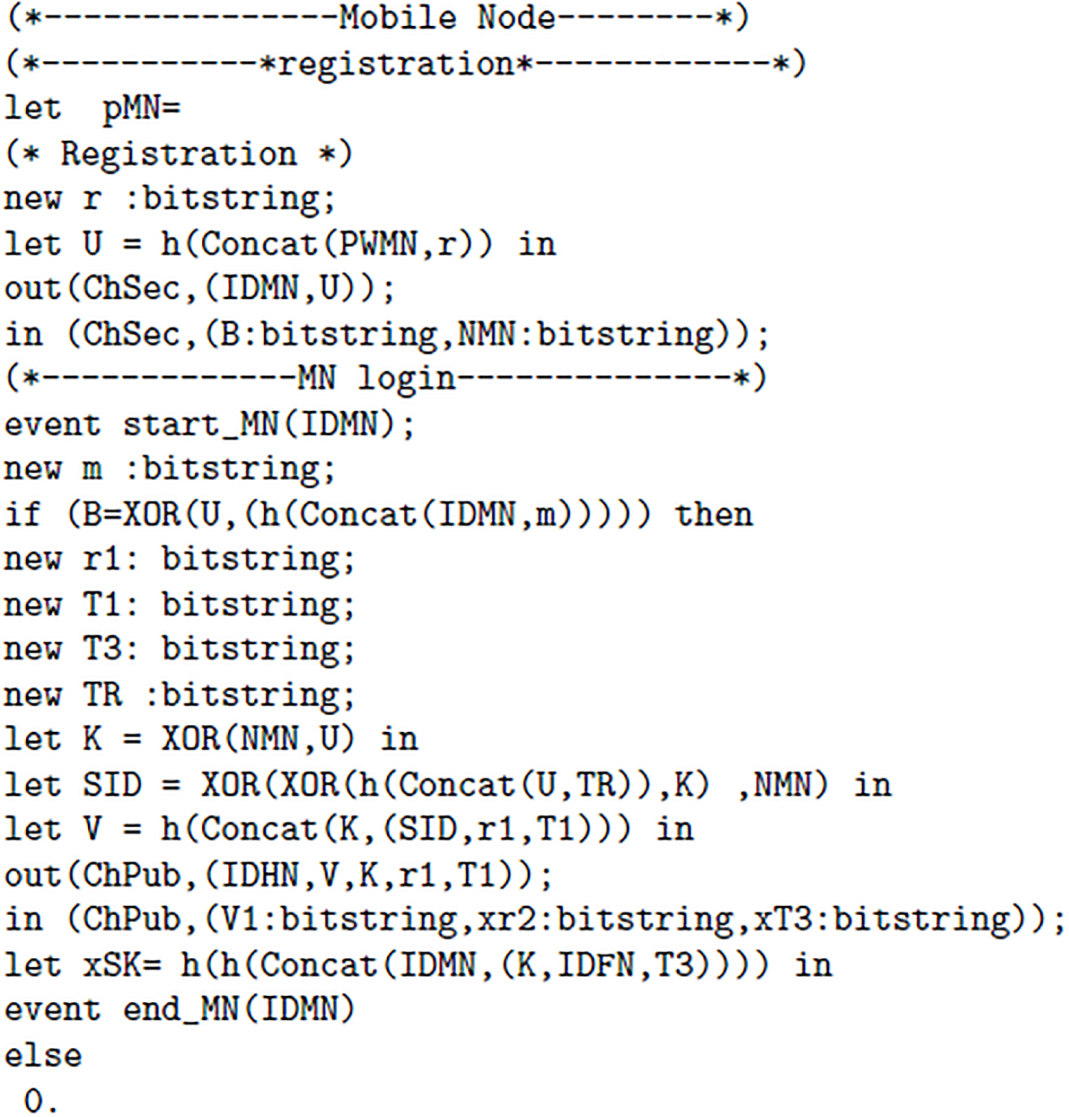
1(c).

The proposed scheme uses two channels one channel “ChSec” is a secure channel which is used between MN and HN in registration phase. Whereas, “ChPub” is called a public or insecure channel. The ChPub is used is login and authentication phase. The Fig 3 1(a) elaborates channels, Constructs and events used in proposed scheme. In Fig 4 1(b) following authentication properties are verified: The query 1 is used to verify whether the session key is secure or not. The query 2 is used for the verification process 1, It determines whether event of Mobile Node(MN) started and terminated successfully or not. The query 3 is used for the verification process 2, It determines whether event of Foreign Node(FN) started and terminated successfully or not. The query 4 is used for the verification process 3, It determines whether event of Home Node(HN) started and terminated successfully or not. Furthermore, we introduced six events, every event represents start and end of each process. Furthermore, Figs 5 1(c), 6 1(d) and 7 1(e) contain full code of three processes(MN, FN and HN)

**Fig 6:**
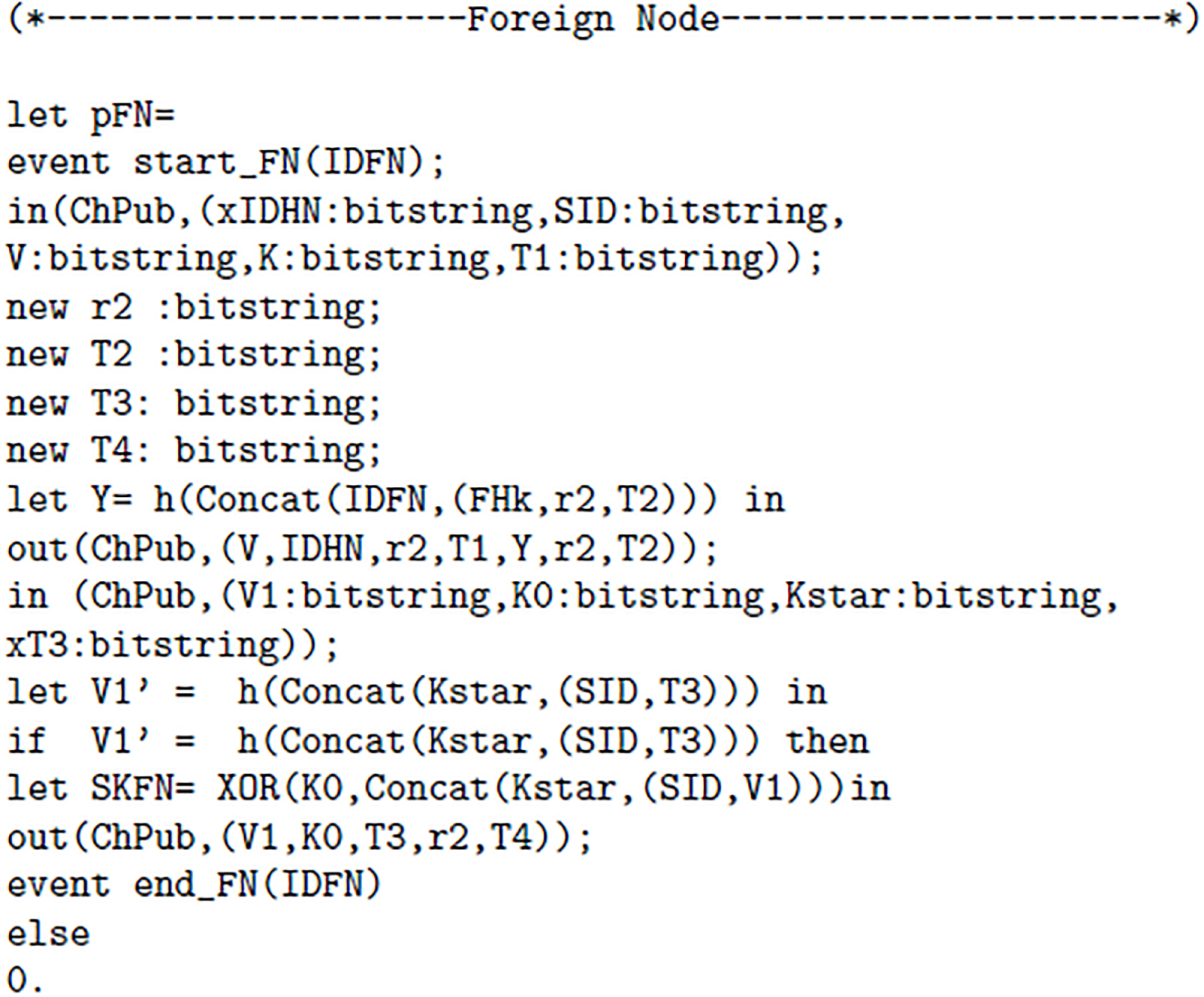
1(d).

**Fig 7:**
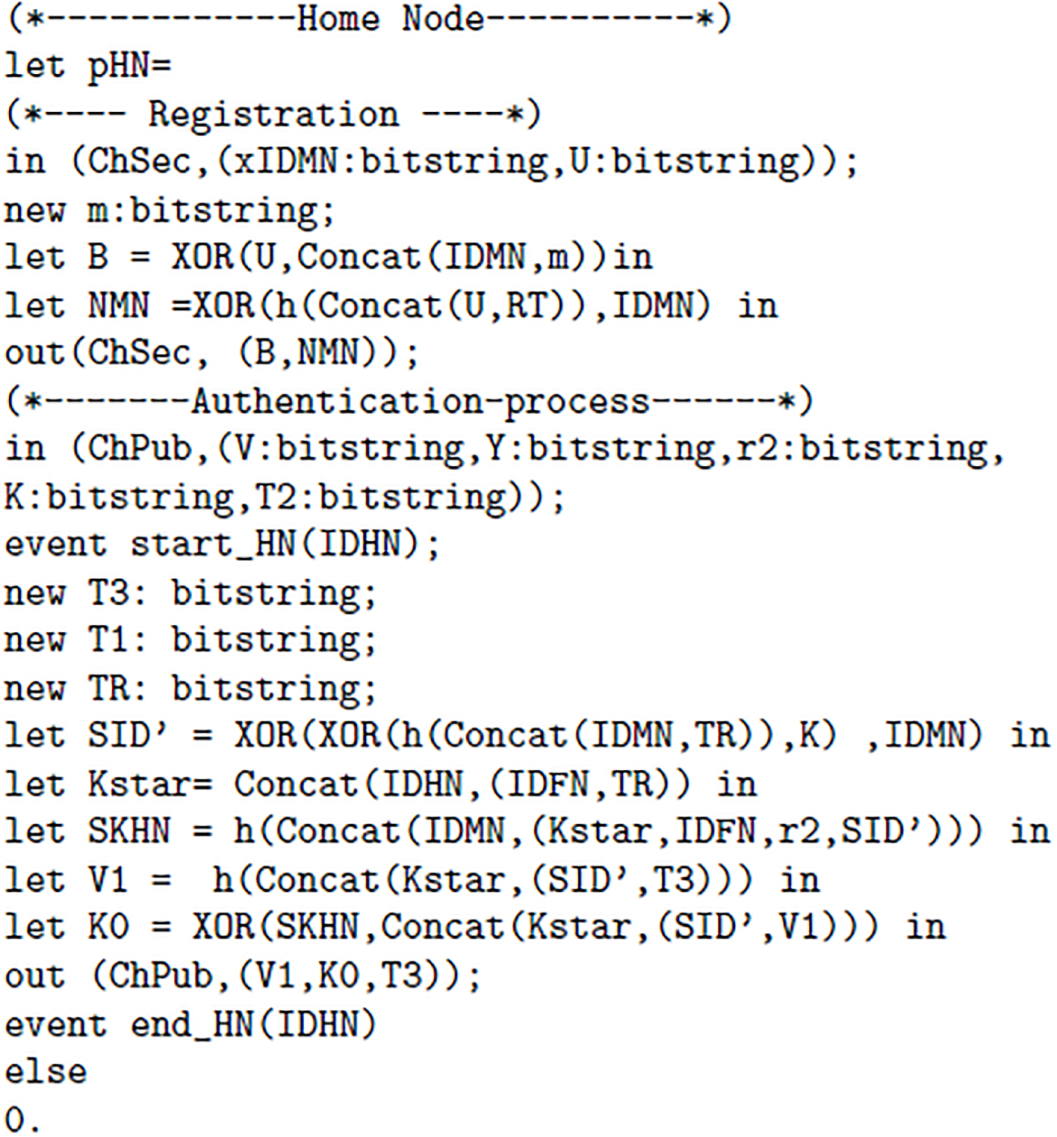
1(e).

**Fig 8:**
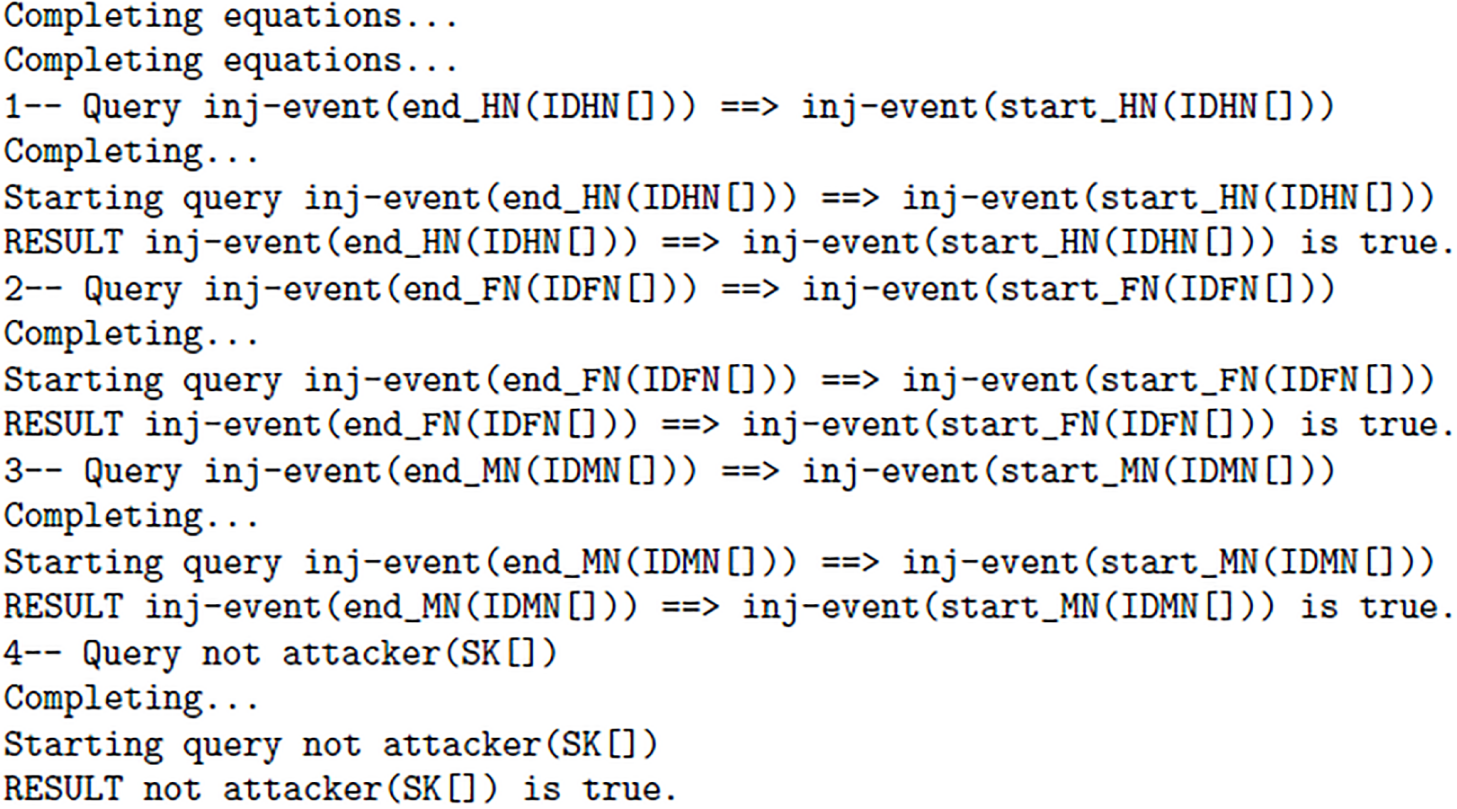
1(f).

The automatic tool ProVerif returns true or false result, When a protocol do not prove the any of the required property then this tool return false result otherwise it returns true result. The results of proposed scheme are shown in Fig 8 1(f) and further elaboration is stated below:

The result 1 demonstrates that process of Home Node(HN) with identity *ID*_*HN*_ has successfully started and terminated The result 2 demonstrates that process of Foreign Node(FN) with identity *ID*_*FN*_ has successfully started and terminated The result 3 demonstrates that process of Mobile Node(MN) with identity *ID*_*MN*_ has successfully started and terminated The result 4 presents the attacker does not access the session-key(*SK*). However, all results demonstrates that the proposed scheme preserves the secrecy and authentication.

All processes (!pHN) | (!pFN) | (!pMN) are executed parallel.

### 5.5 Informal security analysis

This section presents the informal security analysis of proposed scheme, The detailed discussions about different attacks and counter measurements to withstand these attacks are stated in subsections:

#### 5.5.1 Node anonymity

Anonymity is considered a valuable factor in secure authentication protocol, identity of Mobile Node(MN) should not reveal to anyone except the authorized participants. A secure protocol protects personal data and sensitive information of a node so, an attacker/adversary could not analyze any information that can help to breach the security requirements. Our proposed scheme achieves the anonymity requirements because we used strong encryption techniques in our proposed scheme we used hash function in registration phase, *M* = {*ID*_*MN*_, *U*} is sent through secure and reliable channel and we used random numbers that protects our messages. In login-authentication phase lets suppose adversary *A* captures the message *M*_1_ and tires to attain the *ID*_*MN*_ but, identity of Mobile Node is saved in *SID* and *SID* = *h*(*U*‖*R*_*T*_) ⊕ *N*_*MN*_, Adversary A cannot extract SID, we can say that our proposed scheme achieves all requirements of Mobile Node(MN) anonymity.

#### 5.5.2 Node traceability

For a secure protocol traceability is vulnerable issue because, the node traceability may leads to many attacks. Our scheme does not disclose login information or previous history because we used random numbers(*r*_1_, *r*_2_, *m*). Hence in our scheme Mobile Node(*MN*) is untraceable.

#### 5.5.3 Man in the middle attack

In this type of attack the malicious adversary A illegitimately intercepts two parties Communication. The Adversary can capture the sensitive data/information, can send or receive data anytime and may impersonate both parties by pretending Himself/Herself a legal user. In our proposed scheme adversary or attacker cannot perform the Man-In-Middle attack because our proposed scheme provides mutual authentication and endpoint authentication at each side. In our proposed scheme we used the timestamps of each participant with every message {*M*_1_, *M*_2_, *M*_3_, *M*_4_} first time difference is checked at each end if time difference is valid then session begins else more we used random numbers so adversary A cannot guess any secret nor the adversary can compute the session key in addition, proposed scheme provides fair *SK* establishment. Thus, Our proposed scheme can withstand the Man-In-Middle attack.

#### 5.5.4 Backward and forward secrecy

Proposed scheme fulfills backward and forward secrecy requirements due to random numbers and freshly generated timestamps(T), with every new session random numbers and timestamps are freshly generated. So, if current communication keys are revealed to some malicious user, it is not possible to predict previous or future communication key with current keys. the Adversary A can neither generate same random number nor A can generate fresh timestamps. Hence, Adversary A may not compute the *SK*. Therefore we can say that our proposed-scheme accomplishes backward/forward secrecy.

#### 5.5.5 Replay attacks

In replay attacks the malicious user repeats or delays the transmission. There are three participants in Global-Mobility-Networks *MU*, *FNandHN* who authenticate each other and four messages are transmitted among them {*M*_1_, *M*_2_, *M*_3_, *M*_4_} over a public channel. Lets assume an adversary A captures the *M*_1_ and try to perform the replay attacks to FN. On *M*_1_ FN compares the timestamps if it is valid then message is accepted otherwise message would be rejected by FN if adversary generates a timestamps *T*_1_ and timestamp comparison becomes true then adversary tries to compute V’ which is impossible for adversary because adversary has no knowledge of values saved in V’ so adversary cannot forge FN. Similarly we used timestamps with all messages *M*_2_, *M*_3_, *M*_4_ and timestamps(TS) comparison at each session also some other comparisons of different values at different sessions so, an adversary cannot replay any message. Furthermore, without knowing *ID*_*MN*_ an adversary is unable to compute the *SK*. Due to following reasons, our proposed-scheme can resist the replay attacks.

#### 5.5.6 Known key attacks

An Adversary A performs known key attacks when he/she finds palintext associated with ciphertext and the malicious attacker simply perform backtracking operations to trace the plaintext. As stated in previous subsections our proposed scheme uses fresh random numbers and timestamps for each sessions the random numbers are freshly generated. Furthermore, all participants create the session key independently. If an attacker gets the previous session key He/She cannot compute recent session key. Hence, the proposed scheme resists the known-key-attacks.

#### 5.5.7 User friendliness

A secure and useful protocol fulfills requirements of a user friendliness, this means to enable a user to freely pick out his/her identity, password. User friendly schemes provide freedom to change or update his/her password to enhance the security and privacy.

Proposed scheme permits the users to select an identity *ID* and password *PW* freely. Whereas, the SC verifies the inputs and correctness. A User may freely generate the nonce and also can change or updates his/her password so password may keep save from attackers and adversaries.

#### 5.5.8 Local user and password verification

To avoid the illegal access proposed scheme provides the password verification in login-authentication phase and also in password change phase. In registration phase the Mobile Node(*MU*) computed *U* = (*PW*_*MN*_‖*r*) and then computes *B* = *U* ⊕ *h*(*ID*_*MN*_‖*m*) where, in login-authentication phase is re-verified locally if $B^{\prime }\overset{?}{=}B$ then the login phase proceeds to next step otherwise session in aborted. So, by using local password-verification we enhanced our proposed scheme more secure.

#### 5.5.9 Insider attacks

Insider attack may defined as malicious network attack that is committed by an authorized person with legal access. In our proposed scheme let’s suppose some insider of Home Node(*HN*) tries to attain the password of Mobile User(*MU*) by registration message *M* = {*ID*_*MN*_, *U*}. The insider of Home Node(HN) can see the message *M* but could not compute the *U* whereas, *U* = *h*(*PW*_*MU*_‖*r*). The user password is concatenated with a nonce and have been hashed with one-way-hash function. Hence, the insider cannot achieve nonce *r* and it is infeasible for any one to compute password from hash value. So, by following assumptions we say that proposed scheme may prevent the insider attacks.

#### 5.5.10 Stolen-verifier attacks

Proposed scheme resist the stolen-verifier-attacks as, the Mobile Node(*MN*) stored the user’s password in encrypted format even the HN and FN cannot get any information about the user password. If SC is stolen then no one can extract the password because password is save in *U* and this value is in encrypted form, adversary cannot alter the password. Hence, proposed scheme can resist the stolen-verifier attacks.

#### 5.5.11 Mutual authentication

Mutual authentication is robust feature of an authentication protocol, which enables the participants of a protocol to mutually authenticates each other at the same time. Proposed scheme furnished all conditions of mutual authentication between participants *MN*, FN and HN.

- *MN* and *HN* Mutual authentication:

In our proposed scheme *MN* authenticates the *HN* by verifying the $V_{1}^{{}^{\prime }}\overset{?}{=}V_{1}$ in step 4 and Home Node(HN) confirms the *MU* by checking $V_{1}^{{}^{\prime }}\overset{?}{=}h(K^{*}∥SID^{\prime }∥K_{0}∥T_{3})$ in step 2 only a legitimate user can compute $V_{1}^{{}^{\prime }}$ where both participants transfer the secret parameter *ID*_*MN*_ with each other also both participants compute the SK mutually so *MN* and FN authenticates each other mutually in proposed scheme.

- *HN* and *FN* Mutual authentication:

Likewise FN and HN authenticates each other in step 3 HN verifies $Y^{*}\overset{?}{=}Y$ where Y is computed by real Foreign Node FN. a Pre-shared key *FH*_*k*_ is used to secure the Y. In step 3 FN is authenticated by HN, afterward session key(SK) is computed mutually so, our proposed scheme provides the mutual authenticity of FN and HN.

- *FN* and *MN* Mutual authentication:

*FN* authenticates *MN* in step 1 by checking $V\overset{?}{=}h(K∥SID∥r_{1}∥T_{1})$ there is *MN*′ *s* timestamp and only a legal *MN* can compute V. So, after the verification of $V\overset{?}{=}V$ the Foreign Node(FN) authenticates *MN*.

#### 5.5.12 Impersonation attacks

Impersonation attack means an adversary may forge a legitimate user by pretending himself/herself a legal user. Adversary/attacker can delete or modify any message in different manners or can forge the other participants by pretending their self a legitimate user. In proposed scheme we withstand the forgery attacks in following ways as stated in subsections:

- *MN* Impersonation attacks:

Suppose the adversary A intercepts the login message *M*_1_ = {*ID*_*HN*_, *V*, *K*, *r*_1_, *T*_1_} in step 1. When session terminates the Adversary A can try to send login message *M*_1_ to FN. When Adversary A transmits login request message M1 the Foreign Node(FN) confirms freshness of *T*_1_ as, timestamps is not fresh the login request will not be accepted by FN. The adversary can generate a new timestamp $\bar{T_{1}}$ and resend $M_{1}=\{ID_{HN},V,r_{2},K,\bar{T_{1}\}}$ with fresh $\bar{T_{1}}$ to FN. FN confirms the freshness of *T*_1_ the freshness comparison may successful this time. For further confirmation FN scrutinizes whether $V\overset{?}{=}h(K∥SID∥r_{1}∥T_{1})$. Here the values of V is not equal to V’ so request will be rejected. Adversary may also try to impersonate in step 4 but due to comparison of V1’ with V1 the adversary will fail to play the impersonation game in each phase.

- FN Impersonation attacks:

In step 2 adversary will try to impersonate the Home Node(HN) by sending message *M*_2_ = {*M*_1_, *Y*, *r*_2_, *T*_2_}. Without knowing the pre-shared key *FH*_*k*_ the adversary cannot impersonate the FN. Moreover proposed protocol also scrutinizes the differentiation of $Y^{*}\overset{?}{=}Y$ in second phase. Furthermore the HN and FN share the SK secretly. The adversary will not be able to impersonate HN or FN by any mean or by any message.

- HN Impersonation attacks:

Proposed protocol can efficiently withstand HN forgery attacks. If the adversary attempts to forge the MN or FN with the message *M*_3_ = {*V*_1_, *k*_0_, K*, *T*_3_} in third phase. In *M*_3_ we used *V*_1_ for local verification hence, the adversary cannot compute the values of *V*_1_. Thus, proposed protocol can easily withstand the HN impersonation in different steps.

## 6 Security requirements and performance analysis

This section presents the requirements analysis and computation cost analysis of our proposed scheme. The first subsection provides the comparison of different security requirements and the second subsection demonstrates computation cost analysis, cost comparison and execution time comparison with other schemes.

### 6.1 Security requirements

To evaluate the different security requirements, this article compares following security requirements with with Yoon et al. [25], Mun et al. [29] and Lee et al. [30] scheme. R1:Node anonymity; R2:Node Traceability; R3:Man-in-the Middle attack; R4:Backward/Forward secrecy; R5:Replay and Dos attacks; R6:Known-key attacks; R7:User friendliness; R8:Local User and Password verification; R9:Insider attacks; R10:Mutual authentication; R11:Impersonation attacks; R12: Efficiency in user authentication; R13:Formal Verification. As shown in Table 2 only our proposed protocol fulfills all security requirements. Furthermore, this article provides user friendliness, mutual authentication and also formally tested with a well-known verification tool ProVerif. The detailed comparison shown in Table 2.

**Table 2:** Security Requirements.

Schemes	R1	R2	R3	R4	R5	R6	R7	R8	R9	R10	R11	R12	R13
Yoon et al.	0	0	1	1	1	0	0	0	0	1	1	1	0
Mun et al.	0	1	0	0	1	0	1	0	1	1	1	0	0
Lee et al.	0	1	1	1	0	1	1	0	1	1	1	0	0
Proposed Scheme	1	1	1	1	1	1	1	1	1	1	1	1	1
1: Provides, 0: Does not Provide													

### 6.2 Computation cost analysis

**Fig 9:**
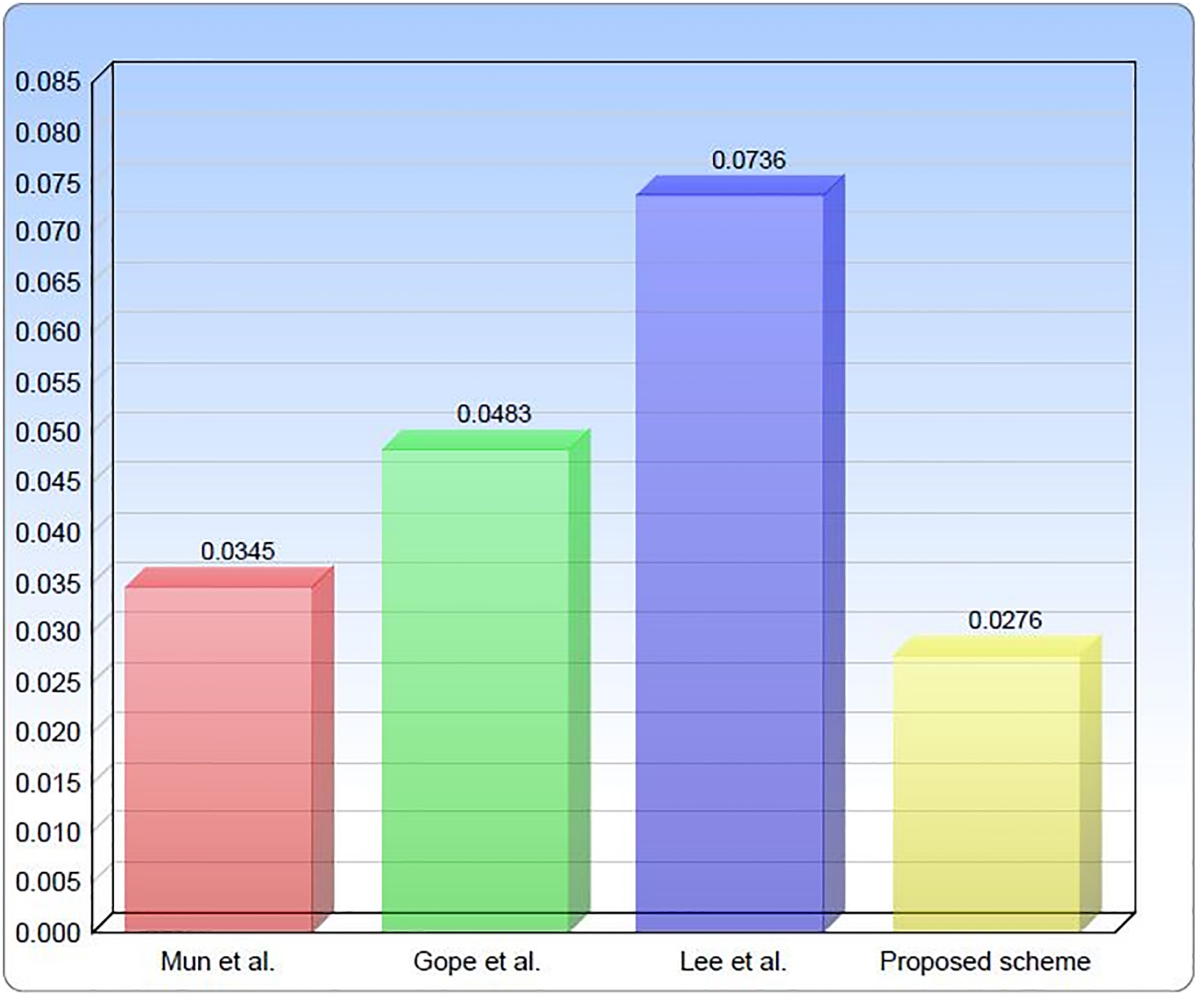
Execution time and performance comparison.

The main focus of the proposed protocol is to safeguard against various security attacks and issues present in the Lee et al. proposal for global mobility networks. In addition, the proposed protocol provides a realistic solution which guarantees reasonable computational cost. In this subsection, a comparison of the protocol with the security protocols of Mun et al. and Lee et al. has been presented based on the number of the state of the art XOR operation, concatenation and hash encryption used in these protocols. The detailed notation guide for each terminology is given in Table 3. For analyzing the proposed protocol in terms of computation cost on the security front, Kilinc and Yanik [32] experimental measurements have been adopted for different encryption operation and functions. According Kilinc and Yanik [32] single Hash encryption utilize 0.0023ms of time in computation. As shown in Table 4, Mun et al. protocol contain 11 times hash encryption, 8-times XOR operation, 4-times Elliptic-curve Point Multiplication(ECMP) and generates the random number 5-times, which in total is 11*T*_*h*_ + 8*T*_⊕_ + 5*T*_*RG*_ + 2*T*_*SE*_ + 4*T*_*PM*_. Similarly, total computation cost of Gope et al. is 21*T*_*h*_ + 16*T*_⊕_ + 3*T*_*RG*_, and the computation cost of Lee et al. protocol is 32*T*_*h*_ + 18*T*_⊕_ + 5*T*_*RG*_, The computation cost of Chaudhry et al. is 8*T*_*h*_ + 6*T*_⊕_ + 3*T*_*RG*_ + 3*T*_*SE*_ + 2*T*_*SD*_. However, total computation cost of the proposed protocol is equal to 12*T*_*h*_ + 10*T*_⊕_ + 4*T*_*RG*_ as shown in Table 4. Moreover, execution time of Mun et al. is 0.0345 and with ECMP it takes total 4.4865ms, the total execution time of Gope et al. scheme is 0.0483ms, the total execution time of Lee et al. scheme is 0.0736ms, Chaudhry et al. scheme takes 0.0414ms and total execution time of our proposed scheme is 0.0276ms the graphical representation of execution time is shown in Fig 9. It is quite clear from the comparison Table 4 and Fig 9 that the proposed scheme has efficient performance. In addition, our proposed scheme satisfies all security requirements using minimum encryption operations and functions. The proposed security protocol successfully attains mutual authentication, node anonymity and have strong resistance against different security attacks.

**Table 3:** Notations guide for computation cost.

*CC*_*MN*_:	Computation-cost of *MN*	*T*_*h*_	One-way-hash operation
*CC*_*HN*_:	Computation-cost of *HN*	*T*_⊕_	XOR operation
*CC*_*FN*_	Computation-cost of *FN*	*T*_*RG*_	Random number generation
*CC*_*total*_	Total Computation-cost	*T*_*PM*_	Elliptic curve Point Multiplication
*T*_*SE*_	Symmetric Encryption	*T*_*SD*_	Symmetric Decryption

**Table 4:** Computation Cost.

Schemes	*CC*_*MN*_	*CC*_*HN*_	*CC*_*FN*_	*CC*_*Total*_
Mun et al.	3*T*_*h*_ + 2*T*_⊕_ + 2*T*_*RG*_ + 1*T*_*SE*_ + 2*T*_*PM*_	5*T*_*h*_ + 4*T*_⊕_ + 1*T*_*RG*_	3*T*_*h*_ + 2*T*_⊕_ + 2*T*_*RG*_ + 1*T*_*SE*_ + 2*T*_*PM*_	11*T*_*h*_ + 8*T*_⊕_ + 5*T*_*RG*_ + 2*T*_*SE*_ + 4*T*_*PM*_
Gope et al.	6*T*_*h*_ + 4*T*_⊕_ + 1*T*_*RG*_	10*T*_*h*_ + 8*T*_⊕_ + 1*T*_*RG*_	5*T*_*h*_ + 4*T*_⊕_ + 1*T*_*RG*_	21*T*_*h*_ + 16*T*_⊕_ + 3*T*_*RG*_
Lee et al.	12*T*_*h*_ + 11*T*_⊕_ + 3*T*_*RG*_	10*T*_*h*_ + 3*T*_⊕_ + 0*T*_*RG*_	10*T*_*h*_ + 4*T*_⊕_ + 2*T*_*RG*_	32*T*_*h*_ + 18*T*_⊕_ + 5*T*_*RG*_
Chaudhry et al.	5*T*_*h*_ + 2*T*_⊕_ + 2*T*_*RG*_	3*T*_*h*_ + 4*T*_⊕_ + 0*T*_*RG*_ + 2*T*_*SE*_ + 1*T*_*SD*_	1*T*_*h*_ + 0*T*_⊕_ + 0*T*_*RG*_ + 1*T*_*SE*_ + 1*T*_*SD*_	8*T*_*h*_ + 6*T*_⊕_ + 3*T*_*RG*_ + 3*T*_*SE*_ + 2*T*_*SD*_
Proposed Scheme	6*T*_*h*_ + 4*T*_⊕_ + 2*T*_*RG*_	5*T*_*h*_ + 5*T*_⊕_ + 1*T*_*RG*_	1*T*_*h*_ + 1*T*_⊕_ + 1*T*_*RG*_	12*T*_*h*_ + 10*T*_⊕_ + 4*T*_*RG*_

## 7 Conclusion

This article scrutinized Lee et al.’s authentication scheme. It has been disclosed that Lee et al. scheme suffers with different security weaknesses. We propose a lightweight and secure two-factor authentication protocol, based on lightweight cryptographic primitives functions such as XOR operations, one-way hash(owh) and concatenation operation. The formal protocol Verification is tested with ProVerif a well known automated tool that confirms the correctness of the proposed scheme and informal security analysis demonstrates that the proposed scheme can withstand different attacks. Security comparison and performance analysis show that the proposed scheme is resistant against all possible attacks and it has very efficient performance making it suitable for practical environment.
